# Charcoal production in the tropical woodlands of southern Mozambique leads to land cover changes—the case of Combomune

**DOI:** 10.1007/s10661-025-14514-4

**Published:** 2025-09-16

**Authors:** Stélio Tchuquela Mabutana, Sverker Molander, Patrik Klintenberg

**Affiliations:** 1 Division of Environmental Systems Analysis, Department of Technology Management and Economics, Chalmers University of Technology, Gothenburg, Sweden; 2 Department of Mathematics and Informatics, Faculty of Science, UEM–Eduardo Mondlane University, Maputo, Mozambique; 3 Department of Spatial Planning, BTH-Blekinge Institute of Technology, Karlskrona, Sweden

**Keywords:** Supervised classification, Landsat, Sentinel-2, Charcoal, Multi-layer perceptron (MLP), Mozambique

## Abstract

This study, conducted in Combomune, Mozambique, investigated land use and land cover changes (LULCC) from 2002 to 2021 and their causes, utilizing Landsat satellite imagery and the land change modeler (LCM). The research mapped vegetation changes and analysed identified explanatory variables. Sentinel-2 imagery was employed to identify charcoal production sites during 2016–2021. The findings indicate significant landscape transformations, with a 20.4% reduction in open forest area (approx. 126,000 ha) and an increase of 22.2% in Shrub (approx. 138,500 ha). Key explanatory variables include proximity to rivers influencing the transition of the class open forest to shrub and proximity to villages influencing the transition of open forest to agriculture and others. Despite these changes, the agriculture and others class exhibited minimal variation compared to open forest and shrub, likely due to a low agricultural productivity influenced by semiarid conditions and poor soil quality. Sentinel-2 imagery revealed numerous burned areas within open forests, suggesting that charcoal production is the primary driver of forest degradation. This finding is consistent with statements from key informants and previous studies that highlight the role of charcoal production in altering forest structure and reducing biomass. The study underscores the urgent need for sustainable forest management practices to mitigate resource depletion related to charcoal production and enhance community resilience against environmental challenges.

**Supplementary Information:**

The online version contains supplementary material available at 10.1007/s10661-025-14514-4.

## Introduction

Woody biomass is still one of Mozambique’s primary energy sources and represents about 85% of the total energy consumed by households (Matavel & Chaves, 2015). Rural and urban populations mainly use woody biomass for cooking food and heating (Falcão, 2013). Woody biomass, primarily charcoal, is a vital source of income for many people in Mozambique (Mabote, 2011). Charcoal is produced in rural areas, but is mainly consumed in urban areas (Chavana, 2014).

Some studies suggest that the charcoal value chain drives forest degradation and deforestation through intensive and selective wood extraction (Chidumayo & Gumbo, 2013; Hosonuma et al., 2012; Ryan et al., 2014). Others claim that harvesting rates in Africa could significantly exceed regrowth (Baumert et al., 2016; Cuvilas et al., 2010; Woollen et al., 2016), causing severe challenges for humans relying on forest ecosystem services. According to Sedano et al. (2020b), charcoal production in southern Mozambique is the main driver of forest degradation. This is primarily due to selective logging rather than clear-felling, which reduces forest biomass (Woollen et al., 2016), a key indicator of degradation. As forest resources become increasingly scarce, local populations are turning to nonrecommended tree species for charcoal production. This practice is likely to accelerate forest degradation and jeopardise the essential ecosystem services that forests provide to local communities Sedano et al. (2020b). Therefore, understanding both past and present forest extent, along with the impacts of human activities and natural phenomena on forest resources, is crucial for developing mechanisms that support the sustainable development goals (SDGs), particularly SDG 7 (affordable and clean energy) and SDG 15 (life on land) (United Nations, 2015).

Today, satellite data are abundant and provide valuable information about the past and present of land use and land cover (LULC) (Yismaw et al., 2014). Several studies have shown that analysing satellite images is the most effective method of monitoring forests (Karlson et al., 2015a; Nesha et al., 2020). Currently, satellite imagery, particularly from passive sensors, has proven valuable for forest monitoring due to the large volume of data available, both commercially and at no cost, which includes both broad temporal and spatial coverage. Landsat imagery serves as a prominent example. Recent advances in the processing and characterisation of the Landsat archive have significantly improved the ability to map land cover and land use globally with greater precision, higher temporal frequency, and more detailed thematic resolution (Potapov et al., 2022). Potapov et al. (2022), developed a global project that leverages these advancements in Landsat data, to enable annual, multidecadal land monitoring. The project generates critical information for assessing global progress toward sustainable development. The results were promising, producing highly accurate thematic maps that enabled the detection of land cover and land use changes over 20 years. At the continental level, Sarfo et al. (2024) conducted analyses using Landsat data and existing literature to investigate specific drivers and mechanisms of land cover change across subregions of Africa, which can support regional efforts to achieve the sustainable development goals (SDGs). Some land use and cover changes (LULCCs) exhibit distinct geographical characteristics, which have led to the development of various methods tailored to specific case studies. Wu et al. (2020), investigated the spatiotemporal variations of forests in the subtropical wetland ecosystem at West Dongting using monthly Landsat normalised difference vegetation index (NDVI) time series data, detecting forest-related changes with an overall accuracy of 87%. According to Shimizu et al. (2019), combining dense time-series observations from optical and synthetic aperture radar satellites can improve forest monitoring over large areas. Shimabukuro et al. (2014), distinguished selectively logged forests from burned forests using multitemporal image segmentation and classification of Landsat data.

Several remote sensing-based studies have been carried out in Mozambique, detecting LULCC in general, and particularly related to charcoal production with a high level of accuracy. (Ryan et al. (2014), quantified changes in the abundance of woody biomass, using a combination of radar remote sensing and ground surveys to investigate what human activities caused the changes. Mahamane et al. (2017), showed how LULCC affected woodland-based ecosystem services using a probabilistic modelling approach combining Bayesian belief networks, geographic information systems, remote sensing data, field data, and stakeholders expertise. Sedano et al. (2020a) monitored forest degradation in a charcoal production area, applying a change detection method analysing temporal NDVI dynamics of historical Landsat imagery.

However, none of the studies above investigated transitions between LULC classes. According to Eastman (2020), transitions are important for understanding the LULC dynamics in the study area, as the main transitions can be identified, grouped, and modelled with potential explanatory variables. Therefore, this process can enhance our understanding of the dynamics between different LULC classes related to charcoal production.

The causes of LULCC include factors that directly or indirectly influence land dynamics (Zhai et al., 2020). According to Geist and Lambin (2002), factors with a direct impact are termed proximate causes. These are immediate actions at the local level, such as agricultural expansion, that result from intentional land use decisions and directly affect forest cover. In contrast, factors with indirect impacts are referred to as underlying driving forces. These include broader social processes, such as population dynamics or agricultural policies, which support the proximate causes and operate either locally or through indirect influence from national or global levels. Geist and Lambin (2002) synthesised findings from local-scale case studies to develop a broader understanding of the proximate causes and underlying drivers of tropical forest change. They identified four main categories of proximate causes: agricultural expansion, wood extraction, infrastructure development, and other factors. The underlying drivers were grouped into five broad categories: demographic, economic, technological, policy and institutional, and cultural factors. In Mozambique, Sitoe et al. (2012), without distinguishing between direct and indirect factors identified a range of factors contributing to forest change including commercial and subsistence agriculture, population growth, urban expansion, commercial firewood harvesting, charcoal production, uncontrolled fires, and mining activities. Furthermore, changes of forest are often shaped by how local populations respond to livelihood opportunities, for example, factors such as the distance to natural resources, proximity to infrastructure like major roads or urban centres, and access to markets can influence land use decisions. These factors are frequently integrated with thematic maps to better understand and explain the causes of LULCC (Iizuka et al., 2017).

The land change modeller (LCM) is a land planning and decision support tool integrated into the TerrSet software (Eastman, 2020). It has recently been applied to study LULCC and the causes behind the changes because it allows users to analyse land cover change, model relationships with various factors, and simulate future land change scenarios. LCM has been used to assess and project land use and land cover changes, addressing issues of accelerated land conversion and the analytical needs required in land management and biodiversity conservation (Gibson et al., 2018; Pérez-Vega et al., 2012). The LCM integrates factors that contribute to the assessment of historical LULCCs, considering the contribution of each factor in this process (Megahed et al., 2015). The LCM can also predict future LULCC (Zhai et al., 2018), producing change probability maps (Iizuka et al., 2017). Identifying the main causes of LULCCs can contribute to improved policies to mitigate or prevent adverse effects of LULCCs (Zhai et al., 2018). Overall, LCM offers a robust framework for analysing LULCC by integrating both proximate causes and underlying driving forces into the modelling process, thereby enhancing our understanding of land change dynamics and their broader implications.

This study sought to pinpoint significant causes of LULCCs between 2002 and 2021 in Combomune, Mozambique, a significant charcoal supplier to Maputo and Matola’s urban areas. The investigation employed remote sensing techniques utilizing Landsat and Sentinel-2 multitemporal data. Ground observation and interviews with local stakeholders were also conducted. The land cover model (LCM) was used to analyse the data and determine the spatial dynamics of vegetation in the area in relation to chosen anthropogenic and biophysical factors, called explanatory variables. These contributions address gaps in understanding transition dynamics and the assessment of explanatory variables, which are crucial for developing sustainable forest policies. Building on previous research in the field, the study presented here offers three novel insights. Firstly, it uniquely analyses interclass transitions in LULCCs using LCM. Secondly, it systematically links explanatory variables to LULCC dynamics using LCM, providing policy-relevant causality insights. Thirdly, it combines data from Landsat, Sentinel-2, ground observations, and local interviews for validation, enhancing accuracy over single-method approaches (e.g., Karlson et al., 2015b; Wu et al., 2020).

## Materials and methods

### Study area

**Fig. 1:**
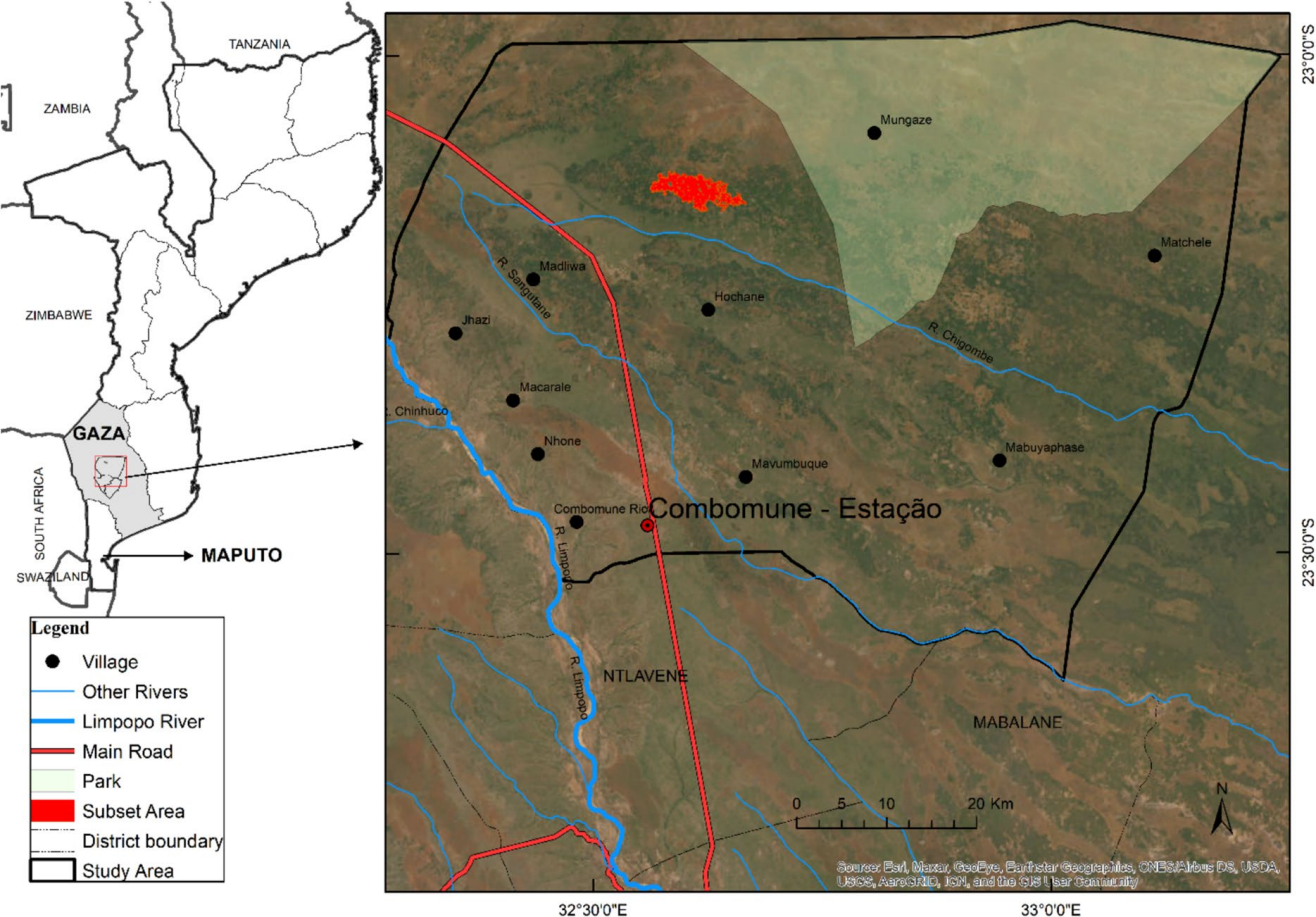
Map of the study area, Combomune, including rivers, roads, and main villages. The area highlighted in red is the smaller area analysed to identify burned areas resulting from charcoal production

**Fig. 2:**
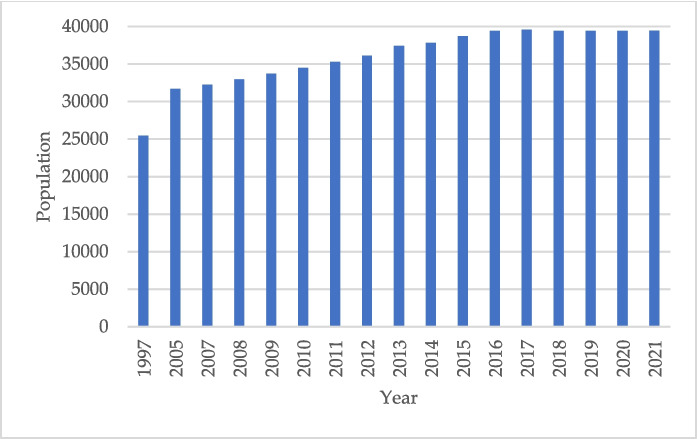
Population in Mabalane district 1997–2021, Statistical Yearbooks of the Mabalane. Data source: (INE, 2022)

The study was conducted in the administrative post of Combomune, in the northern part of the Mabalane district in the province of Gaza in southern Mozambique (Fig. 1). Combomune is a rural region with an area of approximately 511,500 ha. At the time of the latest census 2021, the population of Mabalane district was 39 462 (INE, 2022). The population increased significantly between 1997 and 2005. From that period onward, growth became slower, and in the last seven years, the population has remained relatively stable. This may indicate that, locally, the demand for natural resources has not increased significantly (Fig. 2).

The Mabalane district receives a mean annual precipitation of approximately 500 mm, with an average yearly temperature of 24 °C. About 90% of the precipitation falls during the wet season between October and April (Woollen et al., 2016). The period between May and September is dry and cold, and June and July are commonly dry with clear skies. Woodlands cover over 80% of Mabalane, mainly Mopane woodlands, dominated by the tree species *Colophospermum mopane* (Baumert et al., 2016). Mopane is a dense hardwood species that produces high-quality, slow-burning charcoal. Most of the charcoal produced in Gaza comes from Mopane woodlands. Production and sale of charcoal is one of the main economic activities in the district, along with low-intensity rain-fed agriculture and animal husbandry under a communal grazing system (Baumert et al., 2016).

Several studies have identified the Mabalane district as an area of significant charcoal production from where charcoal is supplied to the two biggest cities of southern Mozambique, Maputo, and Matola, where the charcoal is consumed (Baumert et al., 2016; Chavana, 2014; Malate, 2017; Smith et al., 2019; Zorrilla-Miras et al., 2018). Mabalane has been a significant supplier of charcoal since 2000 (Vollmer et al., 2017). The production has gradually moved from the south to the north of the district as the woody resources become scarcer (Woollen et al., 2016). Sedano et al. (2020b) showed that the pattern of forest degradation in Mabalane district follows the distribution of the mopane woodlands, progressively moving in two directions first, South–North and then West–East outwards from the district's main villages and following the main roads.

In 2001, the administrative post of Combomune was situated at the margin of the charcoal production area and was primarily unaffected by land use/land cover change (LULCC). Since then, charcoal production has intensified and spread into the area. Several locations experienced increased charcoal production during the latter part of the study period (Woollen et al., 2016), motivating further investigation by this study.

### Data acquisition

**Fig. 3:**
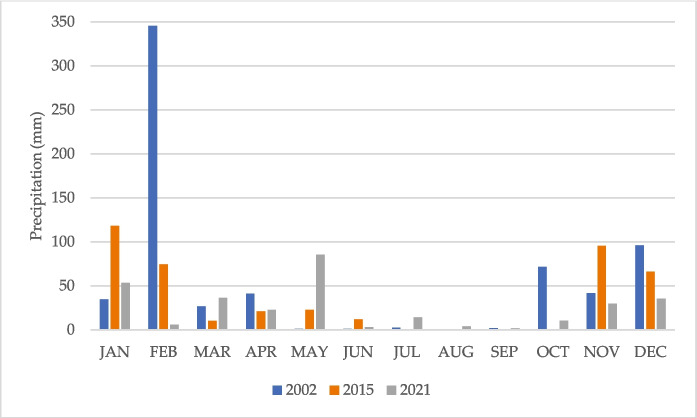
Monthly average precipitation recorded in Combomune for the years 2002, 2015, and 2021. Data source: (NASA, 2021)

**Fig. 4:**
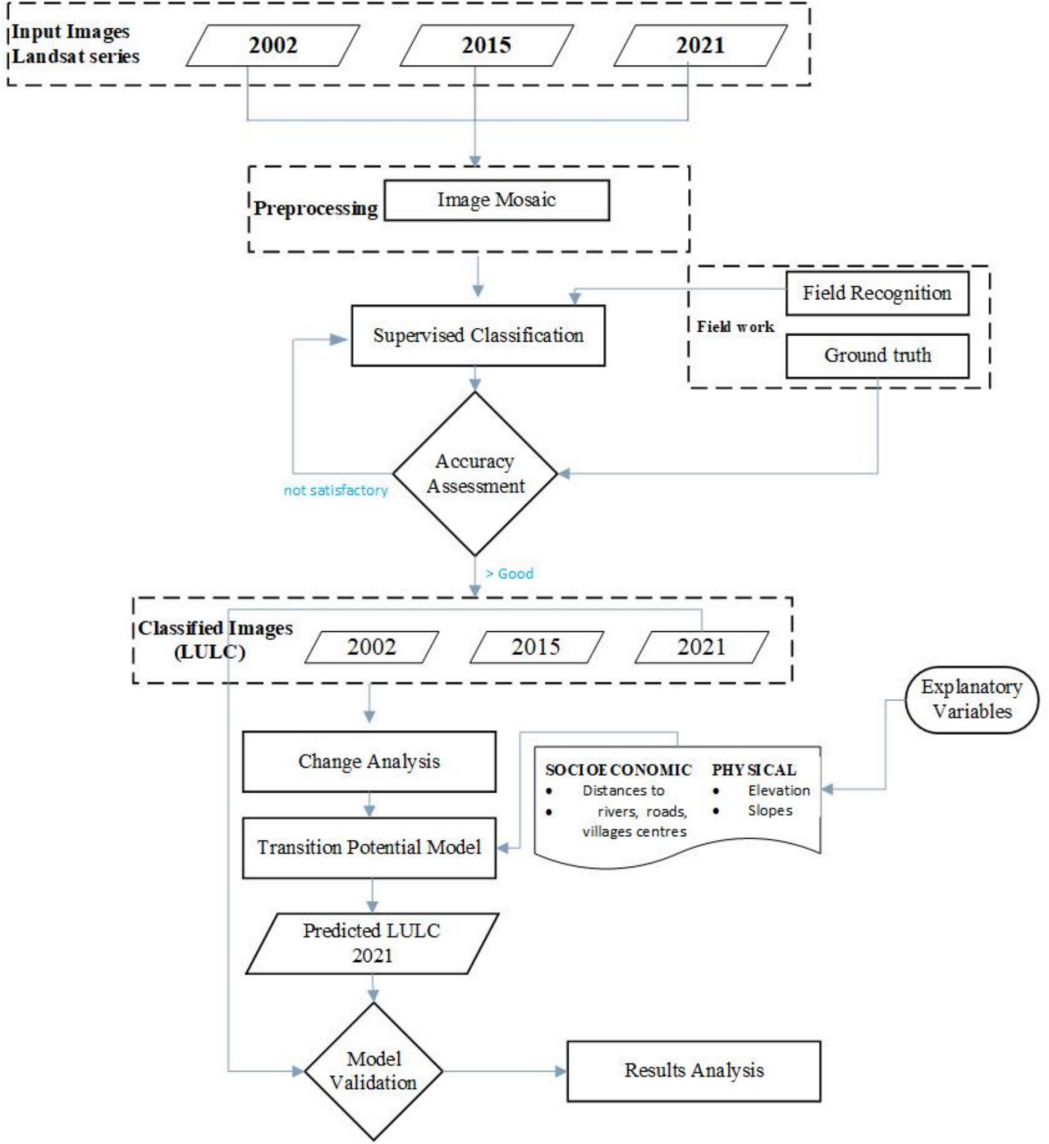
Flowchart presenting the general methodology of the multitemporal part of the study

The vector data (rivers, water bodies, national parks, villages, major roads, and administrative boundaries) were acquired through the National Center for Cartography and Remote Sensing (CENCARTA), responsible for producing and disseminating geospatial data in Mozambique. The available data were digitalised by CENCARTA from topographic maps with a scale of 1: 250,000. The raster data used were Landsat multitemporal satellite images with 30-m resolution, advanced spaceborne thermal emission and reflection radiometer (ASTER) global digital elevation model (GDEM) Version 2 with 30-m resolution and Sentinel-2 with 10 m resolution. Landsat and ASTER GDEM 2 were acquired from the Earth Explorer at the United States Geological Survey (USGS). At the time of collection, the images were atmospherically corrected to surface reflectance Level 2 (USGS, 2020). The Landsat data used for the analysis were two Landsat 7 Enhanced Thematic Mapper (ETM +) images and four Landsat 8 Operational Land Imager (OLI) images. All images were acquired in June, recorded with no (0%) clouds (Table 1). According to Mananze (2012), an image recorded during the dry season allows for better distinction of different types of vegetation. The weather conditions were quite similar during the collection period of all images (Fig. 3). All analysis steps for LCM are summarised in the methodological flowchart (Fig. 4).

**Table 1:** Landsat images used for classification

Date of acquisition	Sensor	Path/row
18/06/2002	ETM +	168/076
27/06/2002	ETM +	167/076
14/06/2015	OLI	168/076
23/06/2015	OLI	167/076
14/06/2021	OLI	168/076
23/06/2021	OLI	167/076

To identify where charcoal production occurs, so-called burn spots, Sentinel-2 images were processed on the Google Earth Engine (GEE). Sentinel-2 is a high-resolution, multispectral imaging mission that offers data suitable for evaluating the condition and changes in vegetation, soil, and water cover. The images undergo orthorectification and atmospheric correction to ensure accurate surface reflectance (Gee, 2024). The Sentinel-2 images used cover a subset of the study area of 2021.3 ha (Fig. 1) for six consecutive years, using mean values from October 2016, October 2017, October 2018, October 2019, November 2020, and October 2021.

### On-site observations and local perceptions of LULCC

To allow for supervised classification of the satellite images and to better understand human activities in the study area, fieldwork was performed in 2021 and 2023, when on-site observations of LULC and semistructured interviews with local key informants were conducted. Informants and field guides were selected in collaboration with community leaders and required to have resided in the Combomune area longer than 10 years and have knowledge about the local forestry, such as tree utilisation, names of commercial trees and experience in charcoal production and agriculture.

The first fieldwork was done in August 2021, conducting on-site observations and interviews with field guides. The aim was to familiarise the researchers with the region and accurately define the LULC classes for the satellite data analysis. As the study involved the establishment of a temporal change, additional observations were made using high-resolution images from Google Earth and previous LULC maps from CENACARTA to enhance the understanding of the region throughout the analysis period. Ground control points were collected in 17 instances of dense forest, 17 of open forest, and 18 in shrub (a total of 52 locations). The classes of agriculture and others, sand, and water bodies did not require field observations as they were easily identified directly in the images.

The second fieldwork was conducted in February 2023, involving additional interviews with local informants to focus on their perceptions of the causes of LULCC in the area during the study period. Semi-structured interviews were held with 15 key informants on charcoal production and agriculture across five villages in Combomune. Three of the respondents were community leaders, and twelve were active charcoal producers at the time of the interviews, as indicated by the community leaders. Each interview ranged from 20 to 40 min. The questionnaire used during the interviews is provided in Supporting information. The questionnaire was divided into two sections. The first section aimed to collect sociodemographic data of the participants, including age, gender, marital status, education level, household size, occupation, time of residence in the community, and emigration history. The second section focused on their perceptions of the causes of changes in the region. The interviews were conducted during the weekend when the informants were available. During weekdays, they were occupied with activities that began early in the morning and ended in the afternoon, after which they attended to their household chores. This limited the number of informants, however, during the later interviews, no additional information occurred, and an information saturation was reached.

### LULC mapping and accuracy assessment

The LULC maps were produced through supervised classification of Landsat images using the maximum likelihood method in ENVI 5.3 software. Maximum likelihood is a commonly used method (Norovsuren et al., 2019; Shivakumar & Rajashekararadhya, 2018; Sisodia et al., 2014). Sample selection for classification was performed by drawing polygons on Landsat images for each LULC class through on-screen observation, informed by fieldwork experience and analysis of high-resolution Google Earth imagery. The following classes were identified: dense forest, open forest, shrub, agriculture and others, sand, and water bodies (Table 2). Due to their spectral similarities, some potential classes (villages, transport infrastructure, crops, including seasonal or perennial crops, and fallow land) were difficult to separate from one another in satellite images and were aggregated into one class: agriculture and others. A similar issue was observed at the boundaries between the dense forest, open forest, and shrub classes. In these boundary areas, there are mixed pixels that may be influenced by climatic or atmospheric conditions on the day the images were recorded. This influence may cause these pixels to be assigned to one of the adjacent classes, potentially influencing the detection of change. Although the issue of mixed pixels was not directly addressed in this research, this limitation was minimised by acquiring images recorded in the same season.

Accuracy assessment was done using the overall accuracy, producer’s accuracy, and user’s accuracy. The overall accuracy measures the proportion of correctly classified pixels in the images. Producer's accuracy indicates how well the training set pixels of a given class are classified. User’s accuracy indicates the probability that a pixel classified into a given class represents that class on the ground (Campbell & Wynne, 2011).

**Table:** Table 2

Classes	Description	Google Earth images
Dense Forest	Woody vegetation consistent with the standards of the definition of forest in Mozambique; an area of at least 1 hectare with a crown cover equal to or greater than 10%, and trees greater than 5 meters in height (Mananze, 2012). According to field observations, *Androstachys johnsonii* locally known as Cimbire (local name) is the most abundant tree species in this class.	
Open Forest	Dominantly mopane woodland, characterised by the species *Colophospermum mopane*. Although it is typically composed of homogenous patches, it can also be associated with a mix of other tree and shrub species (FNDS, 2019).	
Shrub	Land with an area greater than or equal to 0.5 hectares, where the occurrence of spontaneous vegetation composed of shrubs or bush formations with a cover degree greater than 25% and height equal or greater than 50 cm is observed (Martins et al., 2016).	
Agriculture and Others	Combination of classes: villages, transport infrastructure, crops, including seasonal or perennial crops, and fallow land.	
Water Bodies	Areas covered by or saturated with water at the time of recording the satellite image.	
Sand	Sandy areas without vegetation	

### LULCC analysis

For data analysis, the LCM was used. The analysis was based on the multitemporal raster images with the same LULC classes, resulting from the classification of the Landsat images of 2001, 2015, and 2021. The classes were assigned integer numbers to facilitate the comparison. For the analysis, a stepwise process was followed (Fig. 4). The process started with change analysis, followed by transition potential assessment, change prediction, and model validation (Hasan et al., 2020; Megahed et al., 2015).

#### Change analysis

Change analysis was conducted to evaluate the LULCC during the study period, focusing on transitions between classes from 2002 to 2015 and from 2015 to 2021. Given the numerous LULC classes, the potential combinations of transitions can be extensive. The key is to identify dominant transitions, which can be grouped and modelled, termed submodels (Eastman, 2020). For this study, an area threshold of 1000 hectares was set for each transition to capture significant changes, excluding transitions below this threshold. The submodels were modelled separately and combined to simulate a future LULC map. The results were presented as maps and a matrix showing the changed areas between two classified images.

Based on the LULCC data, for each pair of years (2002/2015, 2015/2021, and 2002/2021) annual rate of forest cover change was calculated using Eq. 1 proposed by Puyravaud (2003):

1 $$r=\left(\frac{1}{{t}_{2}-{t}_{1}}\right)\times \text{ln}\left(\frac{{A}_{2}}{{A}_{1}}\right)$$

where ${A}_{1}$ and ${A}_{2}$ are the forest cover at time ${t}_{1}$ and ${t}_{2}$, respectively, the unit is percentage per year.

#### Transition potential modelling

After producing the submodels, the potential for a class to transform into another class was determined by creating potential transition maps using historical information from 2002 to 2015 combined with explanatory variables. The identification of explanatory variables was guided by knowledge acquired from the studies previously mentioned in the introduction section, such as Geist and Lambin (2002), Sitoe et al.(2012) and Iizuka et al. (2017). Based on these references, the following explanatory variables were identified within the study area and applied in the present study: altitude, slope, distance to rivers, distance to roads, and distance to villages. All variables were considered static throughout the time series. The multilayer perceptron (MLP) was used to model the change potential from one class to another. MLP is a machine learning algorithm known for its good performance in combining historical maps with explanatory variables to estimate future LULC (Dzieszko, 2014; Hasan et al., 2020; Iizuka et al., 2017; Megahed et al., 2015; Zhai et al., 2016). The relative importance of each variable in explaining the observed LULCC for each submodel is measured using the skill measure, S (Eq. (2) and (3)), ranging from −1 to 1, with values smaller than 0 indicating lesser influence on LULCC and values close to 1 indicating a better fit (Näschen et al., 2019).

2 $$E(A)=\frac{1}{T+P}$$

where *E*(*A*) is the expected accuracy, *T* is the number of transitions in the submodel, and *P* the number of persistent classes.

3 $$S=\frac{(A-E(A))}{(1-E(A))}$$

where *S* is the skill measure, while *A* is the measured accuracy, which accounts for the percentage of correct predictions.

#### Change prediction

A prediction map for 2021 was constructed based on historical changes from 2002 to 2015, transition maps, and a transition probability matrix. A transition probability matrix shows the likelihood of transitioning from one class to another within the observed period (Hasan et al., 2020). The transition probability matrix was created using transition maps with the reference year 2021. All this information was then used to simulate the LULC map for 2021, with the same classes used in the supervised classification: dense forest, open forest, shrub, agriculture and others, sand, and water bodies.

After simulating the LULC of 2021, a model validation was conducted to compare the quality of the 2021 predicted map with the actual 2021 LULC map generated by supervised classification. The result of this process was used to assess how well the explanatory variables can explain the LULCC identified through supervised classification of the Landsat images from 2002, 2015, and 2021, adding complementary information to the previously mentioned skill measure. The validation process used different accuracy measurements called κ coefficients. According to Eastman (2020), these coefficients evaluate the agreement of maps based on the number of pixels in each class and their locations. The κ coefficients used were as follows: *K*_no_ for overall accuracy, *K*_location_ for location accuracy, and *K*_standard_ for quantity accuracy relative to the reference map classes (Araya & Cabral, 2010).

### Mapping of charcoal production sites

To gain further insight into the causes of LULCC, mapping of charcoal production sites was conducted in a part of the study area (red in Fig. 1). Charcoal production sites were identified using Sentinel-2 imagery, specifically the visible bands (B2, B3, and B4) and the near-infrared (NIR) band (B8), all with a spatial resolution of 10 m. According to Chuvieco et al. (2019) and Sedano et al. (2020b), the NIR band is effective for detecting areas affected by charcoal production. This mapping could not be performed using Landsat imagery due to its lower spatial resolution. The identification and quantification of charcoal production sites were essential for better understanding the proximate causes of LULCC in the region.

To identify and quantify burnt spots, for each Sentinel-2 image, a supervised classification was carried out on the GEE platform over the period 2016–2021. Firstly, the open forest class was extracted from a part of the classified 2002 Landsat images, representing the first year of our study period, when the open forest was presumed to be least degraded. The choice of open forest was to minimise errors in charcoal kiln identification, as this class contains an abundance of mopane trees used for charcoal production.

The masked area was classified into two classes: open forest and charcoal kilns, using a supervised classification. The training samples consisted of polygons for open forest and points for burned spots. Points were used to capture the pixels that represent the recently burned areas, which appear black in the NIR band, much darker than the surrounding pixels. A total of 160 points were generated using an on-screen selection, 60 for open forest and 100 for charcoal kilns. Then, 80% of the training samples were randomly selected for the classification, and the remaining 20% were used for accuracy assessment. The random forest classifier was used, which, according to Zhao et al. (2024), is a widely applied algorithm for image classification used for change analysis, environmental monitoring, and land management applications.

## Results

### Land use and land cover change analysis

**Fig. 5:**
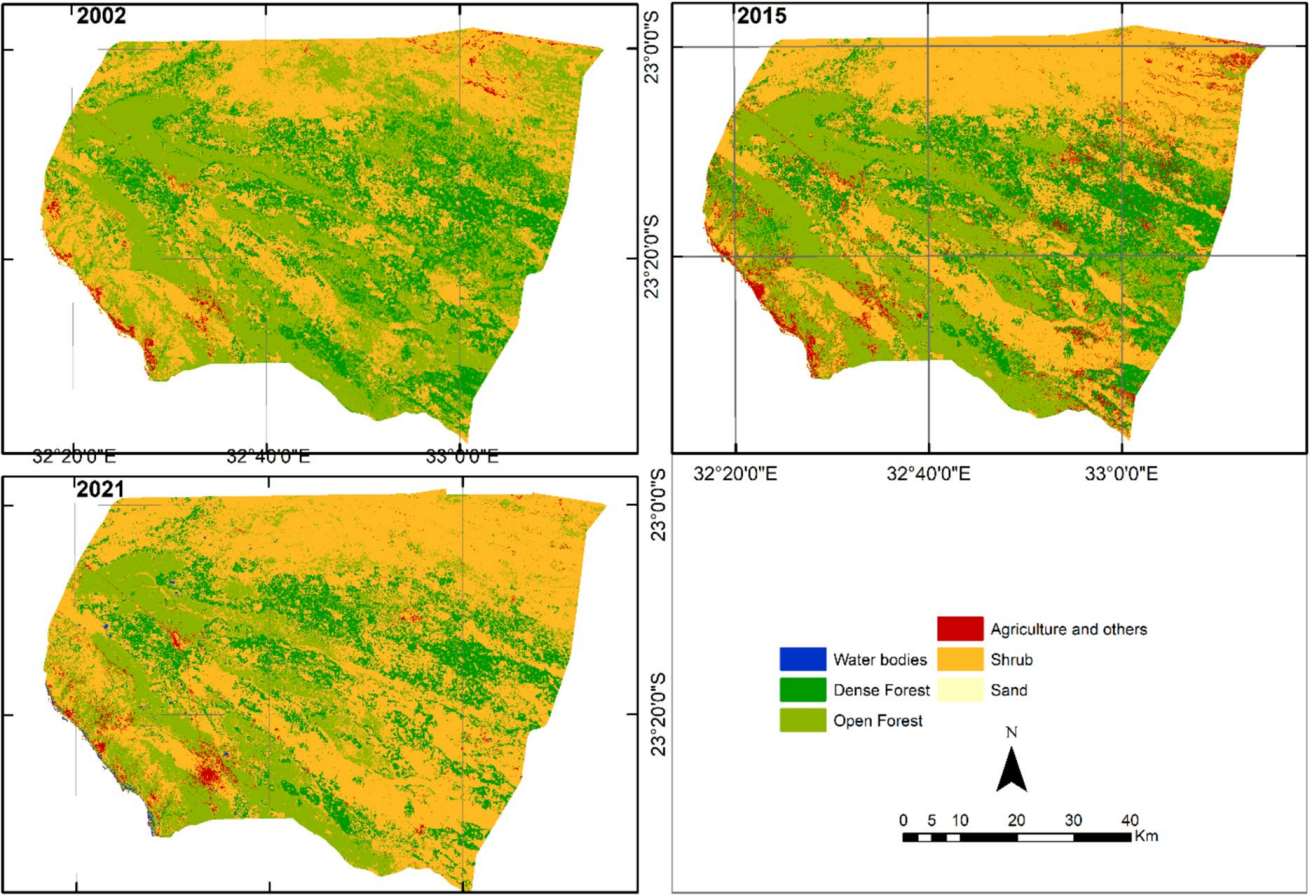
Land use and land cover maps 2002, 2015, and 2021

Figure 5 presents the LULC maps for 2002, 2015, and 2021. The resulting maps are highly accurate, with an overall accuracy of 94.8%, 95.3%, and 97.8%, respectively (see Supporting information). Comparing the results from 2002 to 2021 reveals a decrease in the open forest of 3.7%/year, an increase in shrub of 2.4%/year, and a decrease in the dense forest of 0.7%/year (Table 3). In 2002, the study area was predominantly covered by open forest and shrub, which together accounted for nearly 80% of the total area. Open forest covered 40.1%, while shrub covered 39.4%. Dense forest was the third largest class, covering 19.1%. Agriculture and other land uses, such as sand and water bodies, made up a very small portion of the area, with agriculture and other covering 1.1%, sand covering 0.2%, and water bodies covering a very small area. In 2015, significant changes were observed in the land use and land cover (LULC) classes. The area covered by open forest decreased substantially to 31.9% of the total area, while shrub increased to 45.6%. Dense Forest also decreased, covering 16.9%. Agriculture and other increased to 5.4%. Sand and water bodies remained relatively unchanged. By 2021, shrub covered 61.1% of the total area, showing a continued increase. Open forest occupied 19.7%, indicating a continued decrease. Dense forest covered 16.6%, showing stability from 2015 to 2021. Water bodies covered 0.3% of the study area, indicating an increase likely related to increased precipitation that year (Fig. 3), which filled intermittent bodies of water. The remaining classes experienced only minor changes from 2015 to 2021 (Table 4). It should be noted that agriculture and other increased from 2002 to 2015 and then decreased from 2015 to 2021. This fluctuation is related to the class composition, which comprises classes with spectral similarities that were difficult to discriminate during the classification process, as explained in the methods section. The increase in agriculture and other could be related to the expansion of settlements and rain-fed agriculture. The subsequent decrease is likely due to the abandonment of rain-fed agriculture, which tends to grow vegetation in the following years. This pattern is common in this area, considering the scarce precipitation, as described later in Sect. 3.10.

**Table 3:** Annual rates of LULCC of woody land cover classes

Classes	2002–2015	2015–2021	2002–2021
	**%/year**	**%/year**	**%/year**
Dense forest	− 0.94	− 0.27	− 0.73
Open forest	− 1.77	− 8.01	− 3.74
Shrub	1.12	5.02	2.35

**Table 4:** Area covered by each LULC class for the years 2002, 2015, and 2021

Year	2002		2015		2021	
Classes	**ha**	**%**	**ha**	**%**	**ha**	**%**
Water bodies	14.4	0.0	15.5	0.0	1660.0	0.3
Dense forest	97,827.8	19.1	86,543.2	16.9	85,138.9	16.6
Open forest	205,259.6	40.1	163,020.2	31.9	100,797.8	19.7
Agriculture and others	5612.1	1.1	27,804.2	5.4	7997.6	1.6
Shrub	201,697.2	39.4	233,276.2	45.6	315,175.1	61.6
Sand	996.5	0.2	748.4	0.1	638.2	0.1
Total	511,407.6	100.0	511,407.6	100.0	511,407.6	100.0

**Fig. 6:**
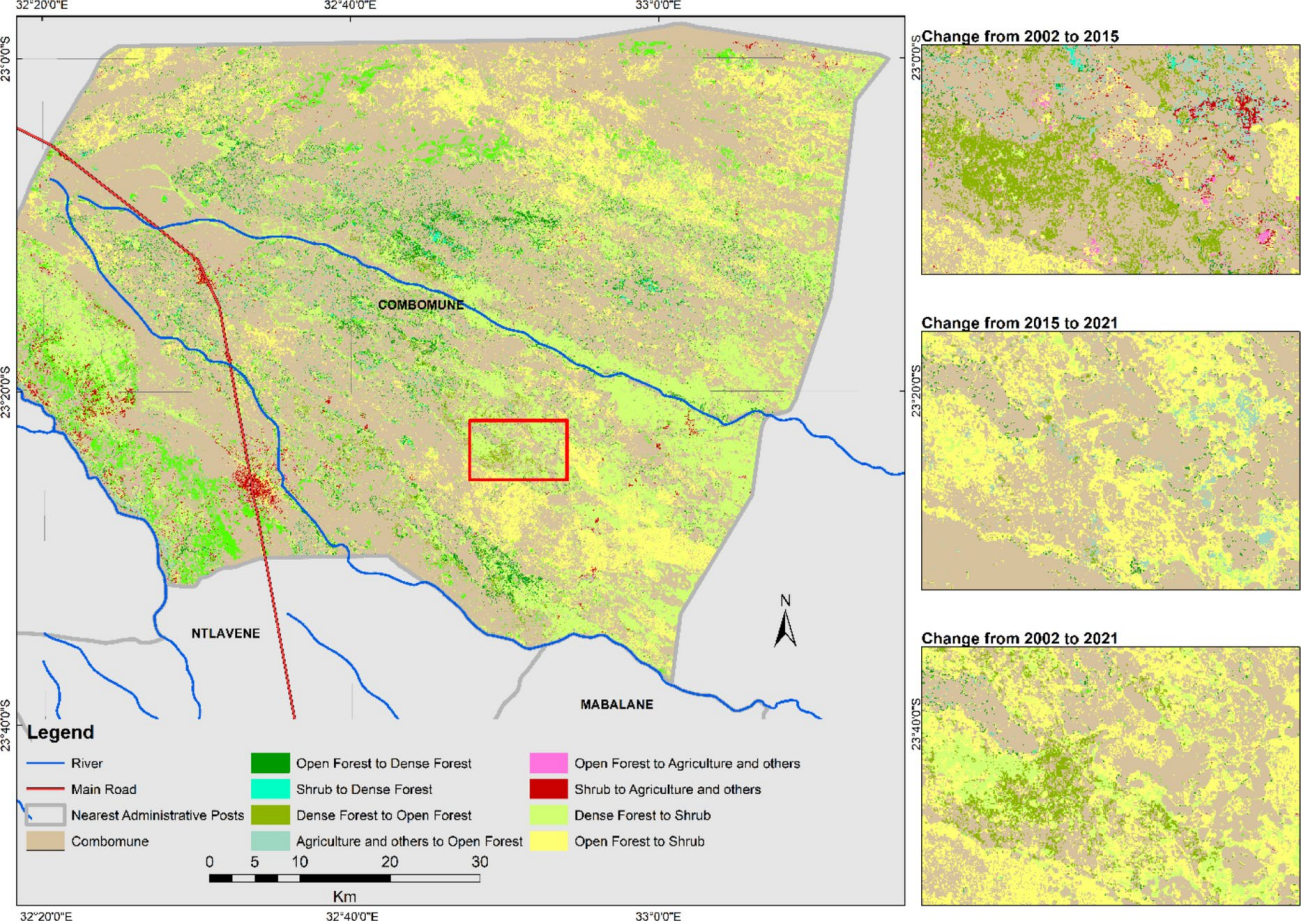
The main transitions during the study period. The large map (left) displays the spatial distribution of the transitions presented in the map legend from 2002 to 2021. The small maps (right) cover the area within the red box in the large map and show the same transitions between the indicated times in the maps

Figure 6 presents spatial transitions between the classes 2002–2015, 2015–2021, and 2002–2021. The corresponding LULC areas in hectares are shown in Table 5, Table 6, and Table 7. The diagonal values in Tables 5, 6, and 7 show the areas of each class that did not change during the given period. From 2002 to 2015 (Table 5), the open forest class experienced a significant net loss of 8.3%, followed by a further net loss of 12.2% from 2015 to 2021 (Table 6). Over the entire period from 2002 to 2021 (Table 7), open forest had a cumulative net loss of 20.5%, primarily due to its transition to Shrub. Dense Forest also experienced a net loss, with a cumulative net loss of 2.3% (Table 7), mainly due to its transition to Shrub. Conversely, the Shrub class experienced cumulative net gains of 22.2% (Table 7) over the study period, with the major contribution coming from open forest. Some of these transitions may be related to pixels with mixed coverage at the boundaries of these tree classes, which may have introduced some uncertainty that was not addressed in the research.

**Table 5:** Land cover change matrix (hectares) during 2002–2015

Classes	Water bodies	Dense Forest	Open forest	Agriculture and others	Shrub	Sand	Total loss
Water bodies	0.5	0	0.6	8.6	0.3	4.3	13.8
Dense forest	0	77,489.2	14,406.8	2700.9	3230.8	0.2	20,338.7
Open forest	0.3	6042.4	123,797	10,318.2	65,088.5	13.1	81,462.5
Agriculture and others	6.1	1.4	393.6	2711.3	2418.8	80.9	2819.9
Shrub	4.2	3010	24,379.5	11,731.7	162,519.5	52.4	39,177.8
Sand	4.3	0.3	42.8	333.5	18.3	597.4	399.2
Total gain	14.9	9054.1	39,223.3	25,092.9	70,756.7	150.9

**Table 6:** Land cover change matrix (hectares) during 2015–2021

Classes	Water Bodies	Dense Forest	Open Forest	Agriculture and Others	Shrub	Sand	Total loss
Water bodies	**12.8**	0	0	1.5	1	0.2	2.7
Dense forest	16	**72,009.2**	258.3	33.3	14,226.2	0.2	14,533.8
Open forest	443.5	10,342.9	**82,571.4**	1507.2	68,141.3	13.8	80,434.9
Agriculture and others	610.6	1496.1	3907.1	**3439.4**	18,203.7	147.5	24,365
Shrub	473.9	1290.8	14,057.6	2875.1	**214,574.9**	4.1	18,701.5
Sand	103.1	0	3.5	141.1	28.1	**472.5**	275.8
Total gain	1647.1	13,129.8	18,226.5	4558.2	100,600.3	165.8

**Table 7:** Land cover change matrix (hectares) during 2002–2021

	**Water Bodies**	**Dense Forest**	**Open Forest**	**Agriculture and Others**	**Shrub**	**Sand**	**Total loss**
Water bodies	**9.1**	0	0.6	2.4	0.9	1.4	5.3
Dense forest	57.2	**73,141.2**	3254.8	163.8	21,208.6	2.3	24,684.4
Open forest	590.9	8832.2	**79,285.9**	2006.2	114,522.1	22.2	125,951.4
Agriculture and others	226.4	9.3	914.7	**1697**	2683.4	81.5	3833.8
Shrub	586.8	3154.9	17,293.1	3910.8	**176,700.3**	51.3	24,996.9
Sand	189.5	1.4	48.8	217.4	59.8	**479.6**	516.9
Total gain	1650.8	11,997.8	21,512	6300.6	138,474.8	158.7	

Note: The transition matrix presents changes in hectares between different classes, including areas that did not change, represented by the values in the shaded diagonal. Transition values are read line by line, e.g., in the first line, 9.1 ha indicates the area classified as Water bodies that didn’t change between 2002 and 2021, and the 0 ha value means no water bodies has changed into dense forest. The net change (positive or negative) is the difference between the totals

### Transition potential modelling and explanatory variables

The most influential explanatory variables were distance to river, altitude, and distance to village, with skill measures of 0.49, 0.39, and 0.18, respectively (Table 8).

**Table 8:** Performance of explanatory variables for each submodel given by the skill measure. Shading indicates more important variables

Variables	Open forest to shrub	Open forest to agriculture and others	Open forest to dense forest	Shrub to agriculture and others	Dense forest to open forest	Dense forest to shrub
All	0.504	0.3841	0.3404	0.2811	0.1136	0.1685
Distance to villages (1)	0.05	0.0072	− 0.0049	0.1789	0.0424	0.0028
Distance to roads (2)	0.0051	0.0072	− 0.0049	−0.0042	0.0306	0.0028
Distance to rivers (3)	0.4899	0.2575	− 0.0049	0.2756	− 0.0414	0.1559
Altitude (4)	0.0051	0.3857	0.2966	− 0.0042	− 0.0049	0.0028
Slopes (5)	0.0051	0.0072	− 0.0049	− 0.0042	0.0932	0.0419

**Fig. 7:**
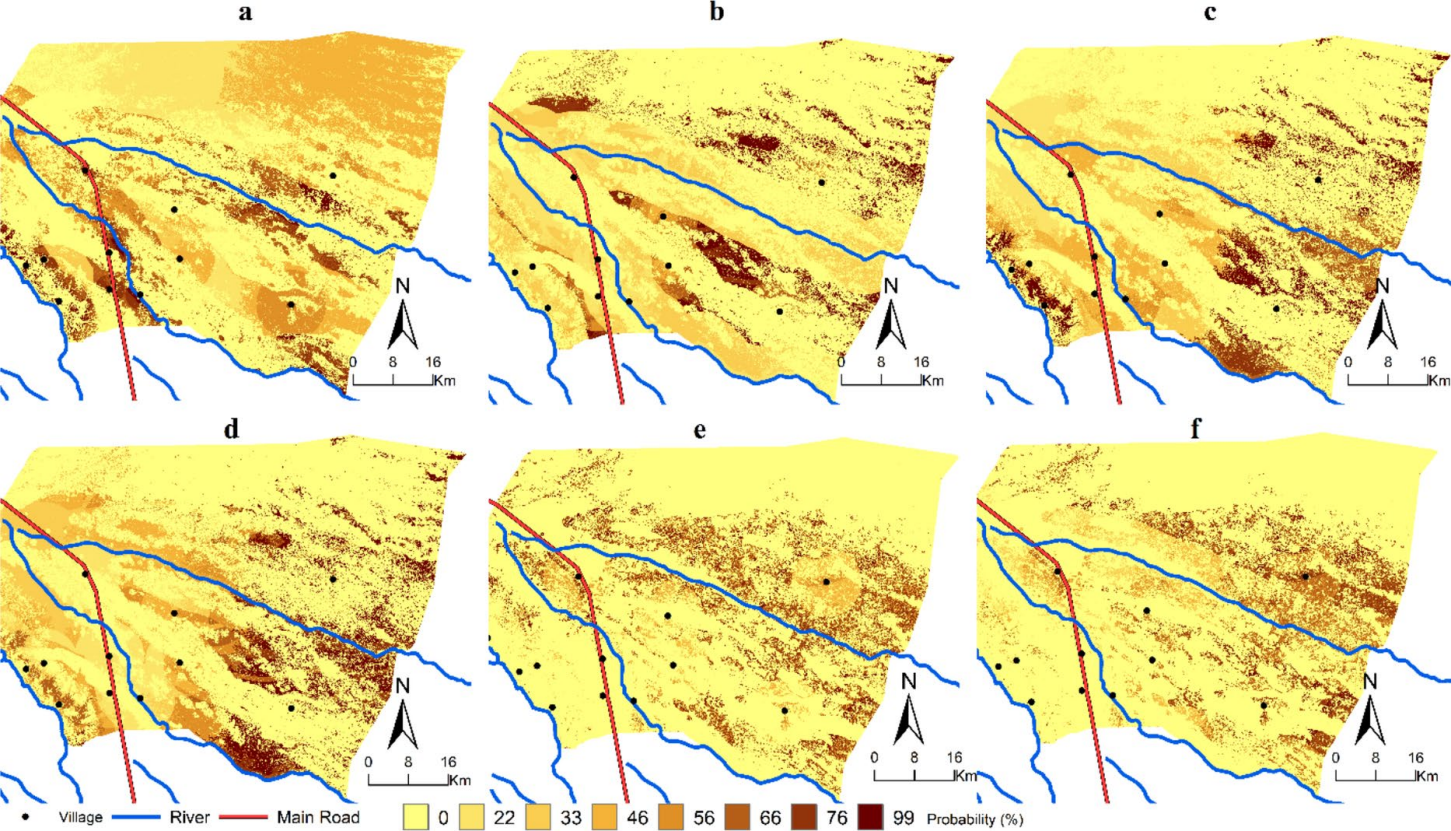
The maps (**a**–**f**) represent the probability of one class change to another. (**a**) Shrub to agriculture and others, (**b**) open forest to shrub, (**c**) open forest to agriculture and others, (**d**) open forest to dense forest, (**e**) dense forest to open forest, and (**f**) dense forest to shrub

According to the transition probability matrix of 2021, dense forest and shrub are the most stable classes, with respective probabilities of 0.89 and 0.88. Open forest and human activities have moderate stability with 0.75 and 0.65, respectively. Water bodies is highly dynamic, with a probability of 0.07. Shrub increased primarily due to decreasing open forest and agriculture and others with the following probability of changes: 0.2 and 0.3, respectively (Table 9). The change probabilities are shown in Fig. 7. The maps show a higher probability of changes from open forest to shrub and shrub to agriculture and others. The probability of change from open forest to shrub is more evident, reaching around 90% in some areas. Probability map a, with changes from shrub to agriculture and others shows high probability of change in some villages of the study area.

**Table 9:** Transition probability matrix of land use land cover for the year 2021

Classes	Water bodies	Dense forest	Open forest	Agriculture and others	Shrub	Sand
Water nodies	0.069	0.001	0.017	0.606	0.000	0.306
Dense forest	0.000	0.893	0.091	0.015	0.000	0.000
Open forest	0.000	0.018	0.748	0.032	0.202	0.000
Agriculture and others	0.001	0.000	0.030	0.650	0.306	0.012
Shrub	0.000	0.007	0.078	0.040	0.875	0.000
Sand	0.004	0.001	0.018	0.213	0.000	0.764

Note: Probability values are read line by line, e.g., in the first line, 0.606 indicates that the class water bodies has 60.6% probability to change for agriculture and others between 2002 and 2015

### Model validation

**Fig. 8:**
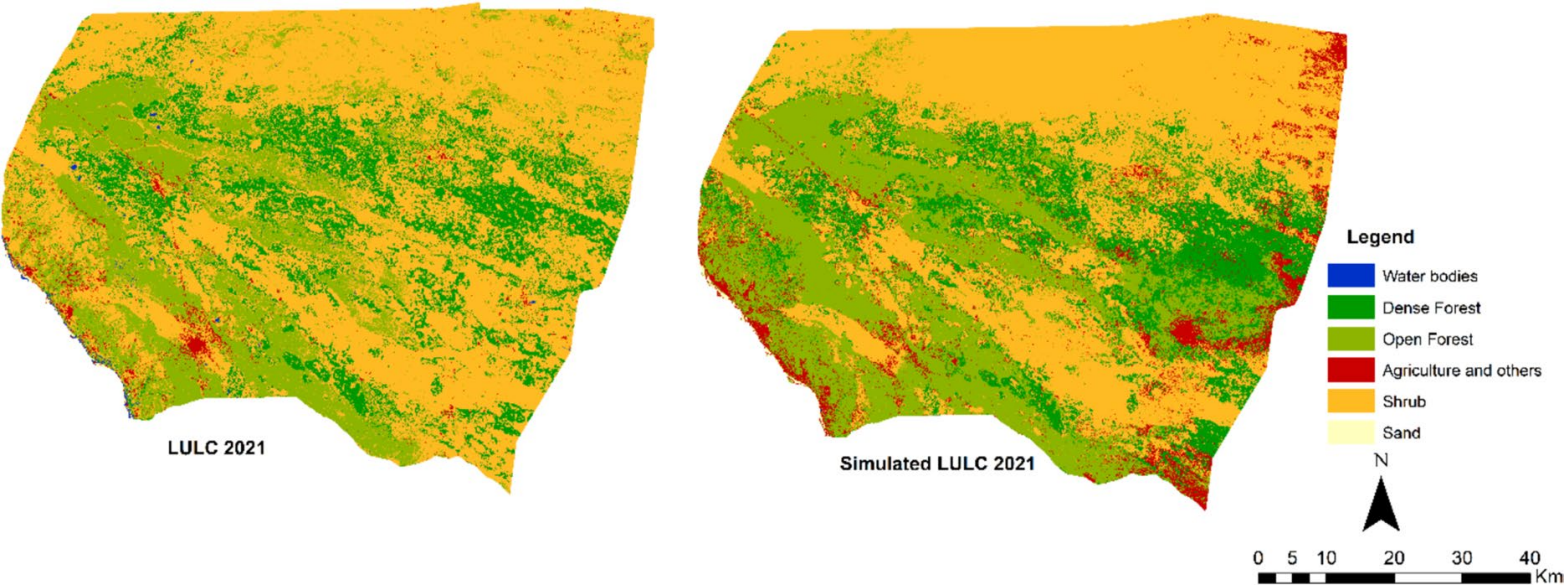
Actual and simulated land use land cover map for 2021

The actual classified image of 2021 and the simulated image of 2021 are presented in Fig. 8. The two maps have good correspondence in terms of locations of pixels representing the same classes, with a *κ* location of 82.4%. The overall accuracy ($\kappa$_no_) is 74.23% (Table 10), which indicates a reasonable agreement between the simulated map and the actual map. Therefore, the applied variables were not sufficient to simulate all the LULC of 2021 using historical changes of 2002 to 2015.

**Table 10:** Compiled results of model validation

Validator	Results %
$\kappa$_no_ (no information)	74.23
$\kappa$_location_ (grid-cell level location)	82.40
$\kappa$_locationStrata_ (stratum-level location)	82.40
$\upkappa$_standard_	69.68

### Burned areas from charcoal production

**Fig. 9:**
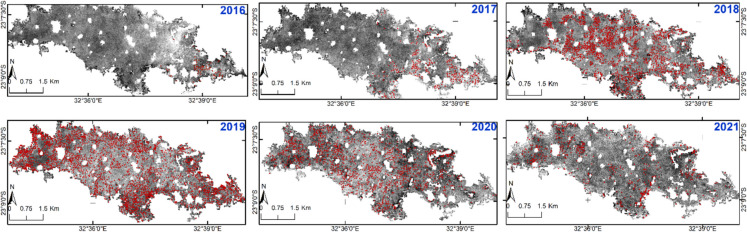
These maps illustrate the distribution of burned spots from charcoal production in the Combomune subset area from 2016 to 2021. The background image visualises the grayscale level of the NIR band from Sentinel-2, with red points representing the burned spots

The intensity of charcoal production across the subset area (Fig. 1) for each year is presented in Table 11. In the image from 2016, 72 burned areas were identified. The highest number of burned areas, 2280, was identified in the image from 2019. These results provide insights into the spatial distribution of charcoal production and its impact on the landscape, showing that charcoal production has taken place in almost all open forest areas in the subset area. The reduction in the number of burned spots after 2019 may be attributed to the decreased availability of preferred trees, which likely motivated the producer to relocate to other areas. Additionally, previously identified burned areas may have regrown vegetation, though they were not identified in the images of subsequent years. The spatial distribution of the burned spots is presented in Fig. 9.

**Table 11:** Number of identified burnt spots in the subset area for the years 2016–2021

Year	2016	2017	2018	2019	2020	2021
Burnt spots	72	260	1477	2280	1082	517

### Local perceptions of the causes of LULCC

During the fieldwork conducted in 2021, the main land uses in Combomune were identified, i.e., livestock rearing, agriculture, charcoal production, and wood collection. At the time of fieldwork, several crop fields appeared neglected. According to the field guides, this neglect was due to fields being prepared for the upcoming rains, which were either delayed or did not occur, leading to vegetation growth and crop damage. Additionally, most water bodies, particularly floodplains, were dry during this time. However, the field guides explained that during heavy rainfall, certain water bodies in the area would accumulate significant volumes of water and could sustain extended periods of water scarcity. Based on the insights from the field guides, our observations, and the literature, the class definitions in Table 2 were applied.

To understand the LULCC and its causes in the study area from the perspective of the local communities, 15 key informants were interviewed. All informants acknowledged that LULCC had occurred in the study area. Charcoal production and small-scale agriculture were said to be the main reasons for LULCC in the study area. Charcoal production involves selective tree cutting, not directly contributing to deforestation but decreasing the availability of the tree species preferred for charcoal production. The key informants stated that the legally permitted tree species for exploitation are rapidly declining in the area and are expected to become scarce within 5 years. The park area (Fig. 1), which contains abundant tree species for charcoal production, is protected by law and cannot be exploited. One of the interviewees, a man of 35 years old, said:

“I started to work in charcoal production in 2004. When I started, the production site was close to my house. When all the desired trees for charcoal production are harvested at one site, we move to another, increasing the distance from home to the production site. Mopane trees are still close to here [his house], but they cannot be harvested as they belong to the park. It's illegal to harvest in the park.”

Key informants said that agriculture in the area is a family-based activity, and they have been using the same fields for crop production for many years. Clearing new fields requires intensive labour, resulting in a limited number of new fields cleared each year. Additionally, the challenging climatic conditions and poor quality of soils limit the potential for high crop yields. The community leader in Chaves, a settlement in the study area, stated that:

“The practice of agriculture is done near the house, not more than 5 km from the homestead. We do not do much yearly clearing of fields for cultivation. Some of the fields we use are inherited from our parents. Opening new fields for agriculture usually happens when the number of people in the household increases. For example, if my son forms a family and wants to cultivate, we can open new fields”.

When asked about restoring forests, the respondents said it is challenging to restore forests and that native trees take a long time to reach adulthood. There have already been several projects for planting fruit trees to generate income, but they have always failed because the climatic and soil conditions are not favourable for this type of tree. One of the interviewees, a community leader in Bairro 1, a settlement in the study area, said:

“The trees that are felled take a long time to regenerate. I do not know how long it takes to reach commercial production. I know it takes a very long time, maybe 70 years.”

## Discussion

This study aimed to identify land use and land cover changes from 2002 to 2021 and their main causes at the administrative post of Combomune in Mozambique, a significant charcoal supplier to Maputo’s and Matola’s urban areas. To do this, four different analyses were done: LULC mapping using Landsat satellite imagery captured years 2002, 2015, and 2021, LCM to identify what explanatory variables drive LULCC in the study area, interviews with local representatives to record their perceptions of causes of LULCC in Combomune, and identification of charcoal production sites in a smaller subarea, using Sentinel-2 satellite imagery for each year from 2016 to 2021 to complement the analysis.

The results of the LULCC analysis indicate that between 2002 and 2021, the area covered by Open Forest decreased by approximately 104,000 ha, which is 20.4% of the original extent. During the same period, the area of Shrub increased by approximately 113,500 ha which is 22.2% of the original extent. This change aligns with the findings of Malate (2017), showing that the actual demand for forest resources exceeds the licensed volume in Combomune, which consequently may put the Combomune community at risk of resource shortage and increasing poverty due to unsustainable forest exploitation.

### Proximate causes influencing LULCC

This study used LCM to assess to what extent selected explanatory variables contribute to LULCC in Combomune. The LCM analysis was also used to create a prediction of LULC for the year 2021, in order to validate the model, but it did not extend to predict future LULC beyond 2021. The analysis of explanatory variables revealed that “distance to rivers” and “distance to villages” were the most influential factors explaining the LULCC from 2002 to 2021. Distance to rivers influenced the transitions from open forest to shrub, reaching a skill measure of 0.5. This spatial relationship is evident in the Fig. 7, illustrating a higher probability of LULCC near rivers and villages. The increased likelihood of change near rivers may be attributed to agricultural clearance activities along riverbanks. This finding is consistent with Malate (2017), who reported that approximately 39% of the population in Combomune engages in agriculture near the Limpopo River due to favourable climatic conditions and better access to water, while the remaining 61% practice rainfed agriculture. Then, distance to villages influenced the transition from open forest to agriculture and others reaching a skill measure of 0.2. The increased likelihood of changes near villages may be attributed to rainfed agriculture. Key informants indicate that this activity typically occurs within a 5-km radius of the villages, explaining the elevated probabilities of changes observed around them. However, Distance to roads was not a significant explanatory variable. This can be attributed to an insufficient coverage of the network of smaller roads and paths, used for charcoal transport to main roads, in the digitised maps.

The LULC class agriculture and others, which is a combination of potential classes of villages, transport infrastructure, crops, including seasonal or perennial crops, and fallow land. This class experienced a sporadic change in 2015, which is possibly related to the typical characteristics of this class, that is, highly influenced by climatic conditions. Climatic conditions, erratic rainfall patterns, and poor soil quality have contributed to low agricultural productivity in areas distant from the Limpopo River (Zorrilla-Miras et al., 2018). This dependence on rain for agriculture may have contributed to the abandonment of open lands for crops due to long waiting periods for rain, and consequently, these areas gained vegetation. Field observations support this finding, as no new agricultural fields were identified, and many existing crop fields were neglected. Additionally, key informants stated that crop fields are typically utilised for several years without frequent clearing for new cultivation areas. Visual image inspection indicates no village sprawl in Combomune, which aligns with data (Fig. 2) from INE (2022), showing that Combomune did not experience a population growth that would have resulted in an increase in the class Agriculture and Others during the analysis period. Distance to rivers, distance to villages, abandoned crops, climatic conditions, precipitation, and soil conditions were the proximate causes identified by the interviews to be the most influential of some LULCC that were observed in the study area, nevertheless were not sufficient to explain the major transition that occurred from open forest to shrub.

### Burned areas from charcoal production and their implications on LULCC

The research explored an additional understanding of the factors that drove the changes between open forest and shrub by analysing the perceptions of the key informants. According to the key informants interviewed, charcoal production is likely to be the primary factor that drove the changes from open forest to shrub. Therefore, an additional analysis was done to investigate this claim. An analysis using the NIR band of Sentinel-2 was done to identify charcoal production sites in a subset of the study area. The analysis detected numerous burned areas attributable to charcoal production. These spots were predominantly observed within the open forest. Given their abundance, it is likely that close to these hotspots no other activity happens, therefore, charcoal production has resulted in a reduction of the open forest and an increase in Shrub. The shrubland typically exhibits lower biomass compared to open forests. Previous research findings indicate that charcoal production primarily contributes to forest degradation rather than outright deforestation (Hosonuma et al., 2012). Degradation is characterised by modification or permanent loss of the forest structure, function, species composition, or productivity due to damaging agricultural and other land uses (Vásquez-Grandón et al., 2018). The mopane tree is highly sought after for commercial purposes and charcoal production (Malate, 2017; Sedano et al., 2021). This species is abundant in unexploited open forests in the study area.

Based on the findings presented here, charcoal production is likely the primary driver of the change from open forest to shrub observed in the study area. The selectivity of the forest harvesting combined with the low growth rate of the preferred wood species leads to forest degradation, which aligns with the identified transition from open forest to shrub. Woollen et al. (2016) consider some villages in Combomune to have reached the peak of charcoal production. This is also in line with Mahamane et al. (2017) who forecasted an extensive loss of mopane due to charcoal production from 2014 to 2025, and Sedano et al. (2020b) who quantified a significant loss of above-ground biomass caused by charcoal production from 2008 to 2018. The analysis done in this study, identifying charcoal production sites in a part of Combomune, supports the findings from previous studies in Mozambique.

### Underlying driving forces and implications of LULCC and sustainable alternatives

Similar to previous studies (Baumert et al., 2016; Chavana, 2014), field research results indicate that the study area is inhabited by several families relying on agriculture for their livelihoods getting into precarious situations during the frequently prolonged droughts. Many of them resort to charcoal production as an alternative due to the unfavourable climatic conditions. Given this scenario, the government should implement mechanisms to support the population in adopting more sustainable agricultural practices, particularly in areas near rivers where water is available. Such measures could help alleviate food insecurity while simultaneously reducing the number of people engaged in charcoal production.

The local population did not experience significant growth during the study period (Fig. 2), suggesting that local demand for natural resources has likely remained relatively stable. In the study area, households primarily use firewood for daily domestic needs, while charcoal is mainly produced for commercial purposes. The primary demand for charcoal appears to originate from urban centres, as Gaza Province, where Combomune is located, is considered a major supplier of charcoal to cities such as Maputo, the capital of Mozambique. In these urban areas, many households rely on charcoal to meet their basic energy needs (De Koning & Atanassov, 2013; Sedano et al., 2021). Continued population growth in these cities may further increase charcoal demand, thereby intensifying pressure on forest resources and contributing to forest degradation (Sedano et al., 2021). One potential solution would be to establish public–private partnerships to invest in sustainable and affordable energy alternatives, promoting modern energy consumption in urban areas and thereby reducing excessive charcoal demand and production.

There is a growing risk that charcoal production may shift from selective exploitation to indiscriminate harvesting due to the scarcity of ideal tree species in accessible areas. This concern was highlighted by key informants and is supported by previous studies (Zorrilla-Miras et al., 2018). It is crucial to develop a monitoring mechanism for areas where charcoal production has already occurred to prevent indiscriminate deforestation and ensure forest regeneration. Furthermore, the government should encourage research on accelerating the recovery of native tree species, as reported by the key informants, as previous reforestation projects have largely failed due to the climatic conditions of the region. These strategies should be analysed and implemented in an integrated manner, involving the entire charcoal value chain to ensure greater effectiveness.

### Study limitations and recommendations for future research

This study presents certain limitations in discriminating LULC classes due to the complexity of the study area. A major challenge was the presence of mixed pixels, requiring more detailed analysis to refine the number of LULC classes. Another crucial aspect is the need for specific mapping of areas burned for charcoal production, distinguishing between legally licensed production and illegal production. This mapping process should implement a near-real-time remote monitoring system to significantly reduce the time and resources needed for field inspections. This is particularly important given the vast extent of the region and the financial and human resource constraints that often hinder effective on-site monitoring (Baumert et al., 2016). The government and private sector partners should actively seek investment opportunities in the energy sector. However, these initiatives must be accompanied by in-depth studies assessing the economic viability and social acceptance of introducing technologies, more efficient both in charcoal production and consumption. The successful implementation of such technologies should be supported by continuous capacity-building and monitoring of the communities involved.

## Conclusions and recommendations for policy and future research

This study, conducted in Combomune, Mozambique, aimed to identify LULCC from 2002 to 2021 and their main causes. The study utilised various methods, including Landsat satellite imagery for mapping, LCM for analysing explanatory variables, and Sentinel-2 imagery for identifying charcoal production sites. The findings reveal significant transformations in the landscape. Over the study period, there was a notable decrease in open forest area by approximately 126,000 ha (20.4% of its original extent), while shrub increased by approximately 138,500 ha. The analysis identified “distance to river” and “distance to village” as key factors influencing transitions from open forest to shrub and open forest to agriculture and others, respectively. Additional analysis of Sentinel-2 imagery detected numerous burned areas in the open forests. This suggests that charcoal production is the primary proximate driver of forest degradation, resulting in decreased open forest and increased abundance of shrubland in the study area.

This study underscores the need for coordinated action between the government and its public and private partners to intervene directly or indirectly in the charcoal value chain. Key areas for intervention include promoting sustainable agriculture to gradually replace rainfed farming, investing in alternative technologies for energy production and consumption, strengthening forest remote sensing monitoring, and developing inclusive policies that support local communities. Reforestation efforts must consider the challenges posed by climate variability, while governance structures should be enhanced to improve the regulation of charcoal production.

Future research should focus on refining remote sensing image classification techniques to tackle the issue of mixed pixels. Additional analysis of the charcoal value chain is also needed to assess the economic feasibility of sustainable charcoal certification, evaluate community acceptance of new energy-efficient technologies, and explore the long-term regeneration of native forests. Moreover, a deeper understanding of the relationship between urban energy demand and forest degradation/deforestation could inform policy adjustments aimed at balancing rural livelihoods with the conservation of natural resources.

## Supplementary Information

Below is the link to the electronic supplementary material.

ESM 1 (DOCX 230 KB)

## Data Availability

No datasets were generated or analysed during the current study.

## Declarations

All authors have read, understood, and have complied as applicable with the statement on “Ethical responsibilities of Authors” as found in the Instructions for Authors.
