# Supplementary material for: Charcoal production in the tropical woodlands of southern Mozambique leads to land cover changes—the case of Combomune
Source: Environ Monit Assess. 2025 Sep 16;197(10):1117. doi: 10.1007/s10661-025-14514-4 (PMC12441091; doi:10.1007/s10661-025-14514-4)
Supplement: Supplementary file 1 — (DOCX 230 KB) [file 10661_2025_14514_MOESM1_ESM.docx]

**Appendix A**

1. **Questionnaire I**
2. **Questionnaire II**
3. **Accuracy Assessment**
4. **Questionnaire I: Interview with the community leader**

**Dear Participant,**

As we presented in the letter, this questionnaire is part of a research program in Natural Resources and Environmental Assessment for Modern Energy Transition in Rural Areas. We are focusing on the Land Use and Land Cover Change (LULCC) associated with the use of forest biomass for energy purposes. We would be very grateful if you could help us to better understand the main factors that influence LULCC locally.

Your contribution is important to us, we would appreciate your time and input. Your responses can be anonymous if you want them to be. If you wish, we could quote your comments or only include your name in the list of interviewees, or you can remain anonymous.

Stélio Mabutana (Research team representative).

1. What do the majority of people in the district do for a living?
2. What are the main economic activities in the district?
3. What are the different areas related to different land uses in the district (i.e., agriculture, forestry, plantations, agroforestry, mining, non-timber forest products)?
4. Who are the main actors in this region who are:
5. Contributing to deforestation, forest degradation, and other activities that generate land use and a land cover change?
6. What do you think are the underlying causes of forest degradation/deforestation?

b. Involved in activities such as forest conservation, reforestation, and transitioning from agriculture to agroforestry?

**Additional questions for each actor in *a* and *b***

c. How did this actor become so influential?

d. Why does this actor have so much/little influence?

e. Where do we find this actor?

f. Are they organised in groups/associations, or are they individuals?

1. What is your role in the management of natural resources? What difficulties and challenges do you face?
2. **Questionnaire II: For Actors Involved in Land Use and Land Cover Change**

**Dear Participant**

This questionnaire is part of research in Natural Resources and Environmental Assessment for Modern Energy Transition in Rural Areas. We are focusing on Land Use and Land Cover changes (LULCC) associated with using forest biomass for energy purposes. We would be very grateful if you could help us better understand the main factors that influence LULCC locally.

Your contribution is important to us, we would appreciate your time and input. Your responses can be anonymous if you want them to be. If you wish, we could quote your comments or only include your name in the list of interviewees, or you can remain anonymous.

Stélio Mabutana (Research team representative).

**Section A:** **Section A aims to collect demographic data of the participants.**

Questionnaire nr.____ Date____/____/_____

Village____ Neighbourhood______

1. **RESPONDENT CHARACTERISTICS AND HUMAN ASSETS**

(a) Age of the respondent___________________

(b) Sex o respondent

| Male |  | Female |  |
| --- | --- | --- | --- |

|  |  |  |  |
| --- | --- | --- | --- |
|  |  | |  |
|  |  |  |  |

c) Marital status

d) What is the size of your household? _________

e) Family size by age group and gender

| Age group | Male | Female | Total |
| --- | --- | --- | --- |
| <17 |  |  |  |
| 18-30 |  |  |  |
| 31-50 |  |  |  |
| >50 |  |  |  |

f) What is your occupation? (CHOOSE ONLY ONE THAT APPLIES)

| Farmer |  | firewood collector |  | Charcoal production |  | Other (Specify) |
| --- | --- | --- | --- | --- | --- | --- |
| Agriculture |  | Livestock Production |  | Student |  |  |
| Business |  |  |  | Domestic work |  |  |
| Housewife |  | Construction |  | Professional |  |  |

1. How long have you been working on this activity?

Less than one year______, between 1 to 4 years______, more than four years_____.

1. If you practice in more than one activity, please tell us how much time you dedicate to each one.
2. Still, on the previous question, how are these activities managed, and how often do you practice each activity?
3. What is the highest level of your education?

| No formal education | Primary | Secondary | Postsecondary | Tertiary | Other(specify) |
| --- | --- | --- | --- | --- | --- |
|  |  |  |  |  |  |

1. Ethnic group

|  |  |  |  |  | Others (specify) |
| --- | --- | --- | --- | --- | --- |
|  |  |  |  |  |  |

m) Do you live here? If yes, go to the next question. If not, why did you choose to work here?

n) How long have you lived in this community?

| <10 years | 11-20 years | >20 years |
| --- | --- | --- |
|  |  |  |

o) If less than 20 years in Qn (j), where did you live before (Village/Traditional Authority/District)

. . . . . . . . . . . . . . . . . . . . . . . . . . . . . . . . . . . . . . . . . . . . . . . . . . . . . . . . . . . . . . . . . . . . . . . . . . . . . . . . . . .

p) What was the reason for migration?

| Farming | Marriage | Employment | Others (Specify) |
| --- | --- | --- | --- |
|  |  |  |  |

**Section B: About the land use and land cover changes**

1. Has there been any change in the location where you have been cutting wood/producing charcoal in the last 5 -10 years? Can you describe it?
2. Do you have any preference for tree species to be felled?
3. Is the preferred tree species abundant in the region, or has its availability decreased over time?
4. Are you aware of any negative impact on the environment associated with coal production activity?
5. If there was a project to plant trees for charcoal production, would you be interested in participating?
6. Do you think a project of this nature would be successful if implemented in your region?
7. Do you know where the coal produced here is sold? (Final destination)?
8. Is there any regulation or licensing system for the exercise of your activity?
9. Do you have a license, or have you already applied for it? If not, why?
10. Is your activity responsible for massive changes locally?
11. Which parts of the region have undergone high levels of deforestation and degradation? What are the key drivers of deforestation and degradation in these areas?
12. Which parts of the region have seen forest recovery, reforestation, conservation, or other low emissions development activities?
13. What are the key drivers of improved land and forest management in each area?
14. **Accuracy Assessment**

**Table 1 Confusion Matrix of 2002**

**Overall Accuracy = (41472/4377) = 94.7477%**

| **LULC** | **Water** | **Dense Forest** | **Open Forest** | **Human Activities** | **Shrub** | **Sand** | **Total** |
| --- | --- | --- | --- | --- | --- | --- | --- |
| **Water** | 10513 | 0 | 0 | 0 | 0 | 0 | 10513 |
| **Dense Forest** | 0 | 3140 | 29 | 0 | 0 | 0 | 3169 |
| **Open Forest** | 0 | 10 | 8627 | 9 | 305 | 5 | 8956 |
| **Human Activities** | 0 | 0 | 0 | 1788 | 1470 | 95 | 3353 |
| **Shrub** | 8 | 0 | 172 | 138 | 16370 | 7 | 16695 |
| **Sand** | 0 | 0 | 0 | 51 | 0 | 1034 | 1085 |
| **Total** | 10521 | 3150 | 8828 | 1986 | 18145 | 1141 | 43771 |

**Table 2** **Producer’s (PA), User’s (UA) and Overall Accuracy of 2002**

|  |  |  |  |  |  |  |  |
| --- | --- | --- | --- | --- | --- | --- | --- |
| **LULC** | **Water** | **Dense Forest** | **Open Forest** | **Human Activities** | **Shrub** | **Sand** |  |
| **Total Pixels** |  |  |  |  |  |  |  |
| **UA (%)** | 100 | 99.08 | 96.33 | 53.33 | 98.05 | 95.3 |  |
| **PA (%)** | 99.92 | 99.68 | 97.72 | 90.03 | 90.22 | 90.62 |  |
| **Overall (%)** | 94.7477 |  |  |  |  |  |  |
|  |  |  |  |  |  |  |  |
|  |  |  |  |  |  |  |  |

**Table 3 Confusion Matrix of 2015**

**Overall Accuracy = (41731/43771) = 95.3394%**

|  |  |  |  |  |  |  |  |
| --- | --- | --- | --- | --- | --- | --- | --- |
| **LULC** | **Water** | **Dense Forest** | **Open Forest** | **Human Activities** | **Shrub** | **Sand** | **Total** |
| **Water** | 10520 | 0 | 0 | 0 | 0 | 0 | 10520 |
| **Dense Forest** | 0 | 3149 | 1 | 0 | 0 | 0 | 3150 |
| **Open Forest** | 0 | 1 | 8809 | 10 | 20 | 1 | 8841 |
| **Human Activities** | 1 | 0 | 9 | 1832 | 1816 | 27 | 3685 |
| **Shrub** | 0 | 0 | 9 | 132 | 16308 | 0 | 16449 |
| **Sand** | 0 | 0 | 0 | 12 | 1 | 1113 | 1126 |
| **Total** | 10521 | 3150 | 8828 | 1986 | 18145 | 1141 | 43771 |
|  |  |  |  |  |  |  |  |

**Table 4 Producer’s (PA), User’s (UA) and Overall Accuracy of 2015**

| **LULC** | **Water** | **Dense Forest** | **Open Forest** | **Human Activities** | **Shrub** | **Sand** |
| --- | --- | --- | --- | --- | --- | --- |
| **Total Pixels** |  |  |  |  |  |  |
| **UA (%)** | 100 | 99.97 | 99.64 | 49.72 | 99.14 | 98.85 |
| **PA (%)** | 99.99 | 99.97 | 99.78 | 92.25 | 89.88 | 97.55 |
| **Overall (%)** | 95.34% |  |  |  |  |  |

**Table 5 Confusion Matrix of 2021**

**Overall Accuracy = (16723/17091) = 97.8468%**

| **LULC** | **Water** | **Dense Forest** | **Open Forest** | **Human Activities** | **Shrub** | **Sand** | **Total** |
| --- | --- | --- | --- | --- | --- | --- | --- |
| **Water** | 519 | 3 | 0 | 0 | 0 | 6 | 528 |
| **Dense Forest** | 2 | 2947 | 0 | 0 | 0 | 0 | 2949 |
| **Open Forest** | 1 | 11 | 2753 | 2 | 11 | 2 | 2780 |
| **Human Activities** | 0 | 0 | 0 | 1422 | 223 | 15 | 1660 |
| **Shrub** | 0 | 25 | 6 | 52 | 7829 | 1 | 7913 |
| **Sand** | 0 | 0 | 0 | 8 | 0 | 1253 | 1261 |
| **Total** | 522 | 2986 | 2759 | 1484 | 8063 | 1277 | 17091 |

**Table 6 Producer’s (PA), User’s (UA) and Overall Accuracy of 2021**

| **LULC** | **Water** | **Dense Forest** | **Open Forest** | **Human Activities** | **Shrub** | **Sand** |
| --- | --- | --- | --- | --- | --- | --- |
| **Total Pixels** |  |  |  |  |  |  |
| **UA (%)** | 98.3 | 99.93 | 99.03 | 85.66 | 98.94 | 99.37 |
| **PA (%)** | 99.43 | 98.69 | 99.78 | 95.82 | 97.1 | 98.12 |
| **Overall (%)** | 97.85% |  |  |  |  |  |

**Table 7 Producer’s (PA), User’s (UA) and Overall Accuracy of burned spots from charcoal production**

| **Year** | **LULC** | **Burned spots** | **Mopane** | **Others** |
| --- | --- | --- | --- | --- |
| **2016** | **UA (%)** | 99 | 98 | 99 |
|  | **PA (%)** | 92 | 99 | 73 |
|  | **Overall (%)** | 98 | | |
| **2017** | **UA (%)** | 86 | 98 | 99 |
|  | **PA (%)** | 92 | 99 | 70 |
|  | **Overall (%)** | 98 | | |
| **2018** | **UA (%)** | 98 | 98 | 97 |
|  | **PA (%)** | 96 | 99 | 85 |
|  | **Overall (%)** | 97 | | |
| **2019** | **UA (%)** | 95 | 98 | 99 |
|  | **PA (%)** | 93 | 99 | 92 |
|  | **Overall (%)** | 98 | | |
| **2020** | **UA (%)** | 98 | 97 | 98 |
|  | **PA (%)** | 96 | 99 | 89 |
|  | **Overall (%)** | 97 | | |
| **2021** | **UA (%)** | 87 | 96 | 99 |
|  | **PA (%)** | 92 | 99 | 83 |
|  | **Overall (%)** | 96 | | |
